# RAD001-Adjuvanted Influenza Vaccine Synergizes With Anti–Programmed Cell Death Protein 1 Therapy to Enhance Antitumor Immunity in Programmed Cell Death Ligand 1–Low Murine Lung Cancer

**DOI:** 10.14740/wjon2831

**Published:** 2026-09-04

**Authors:** Hui Lin Ou, Jia Sun, Shi Bo Wu

**Affiliations:** a Ningbo Medical Centre, Li Huili Hospital Affiliated of Ningbo University, Ningbo, Zhejiang 315040, China

**Keywords:** Lung cancer, Cancer immunotherapy, Rad001 adjuvant, Influenza vaccine, Tumor microenvironment, PD-L1–low

## Abstract

**Background:**

Immune checkpoint inhibitors targeting programmed cell death protein 1 (PD-1) have transformed lung cancer therapy, but their efficacy is often limited in tumors with low programmed cell death ligand 1 (PD-L1) expression and immunologically “cold” tumor microenvironments. This study aimed to evaluate the safety and therapeutic efficacy of combining a RAD001-adjuvanted influenza vaccine with PD-1 blockade and to explore its immunological mechanisms in a murine lung cancer model.

**Methods:**

C57BL/6 mice bearing Lewis lung carcinoma (LLC) tumors, characterized by low PD-L1 levels (10.28%), were randomized to receive control, anti-PD-1 monotherapy, or anti-PD-1 combined with either a split influenza vaccine, an MF59-adjuvanted vaccine, or a RAD001-adjuvanted vaccine. Primary endpoints included tumor growth kinetics and survival. Safety was assessed via histopathological analysis of major organs and biochemical analyses. Mechanistic changes were evaluated through immunophenotyping of tumor-infiltrating lymphocytes (TILs) and serum cytokine profiling.

**Results:**

The RAD001-adjuvanted vaccine synergized with anti-PD-1 therapy, resulting in superior tumor growth inhibition compared to all other groups. This synergy was associated with a remodeled tumor microenvironment, showing increased CD8^+^ T-cell infiltration and a higher CD8^+^/regulatory T-cell ratio. Serum analysis revealed a shift toward a Th1-dominant cytokine profile (a trend toward increased interferon-gamma and interleukin-2). Histopathological and biochemical examination confirmed no treatment-related toxicities in the heart, liver, lungs, kidneys, or spleen.

**Conclusions:**

RAD001-adjuvanted influenza vaccination safely enhances the efficacy of anti-PD-1 therapy in PD-L1–low murine lung cancer by promoting a pro-inflammatory immune microenvironment. These findings suggest that strategic vaccine adjuvant selection can broaden the therapeutic reach of immune checkpoint inhibitors.

## Introduction

Immune checkpoint inhibitors (ICIs) targeting the programmed cell death protein 1 (PD-1)/programmed cell death ligand 1 (PD-L1) axis have revolutionized the treatment of advanced lung cancer by reactivating T cell–mediated tumor killing, an effect that is strongly dependent on the level of tumor PD-L1 expression [1, 2]. Nevertheless, the clinical efficacy of this approach is often limited by primary or acquired resistance. Such resistance is frequently associated with an immunosuppressive tumor microenvironment (TME) characterized by insufficient infiltration of tumor-infiltrating lymphocytes (TILs), particularly cytotoxic CD8^+^ T cells, together with the presence of immunosuppressive cells such as regulatory T cells (Tregs), a condition commonly referred to as a “cold” tumor [3]. Converting these “cold” tumors into “hot,” immunologically active tumors has therefore become a major focus of contemporary oncology research. Current strategies to achieve this transformation include combining ICIs with therapies capable of stimulating innate immunity and promoting T-cell infiltration into the TME [4].

The mammalian target of rapamycin (mTOR) is a central regulator of cellular metabolism, growth, and immune responses, integrating signals from nutrients, growth factors, and environmental cues. Increasing evidence indicates that mTOR signaling also plays a critical role in shaping immune cell function, including T-cell differentiation and cytokine production.

RAD001 (everolimus) is a derivative of rapamycin and a well-characterized inhibitor of mTOR, a key regulator of cell growth, metabolism, and immune function. It is widely used in clinical settings for cancer therapy and as an immunosuppressive agent in organ transplantation. In addition to its antiproliferative effects, accumulating evidence suggests that mTOR inhibition may exert context-dependent immunomodulatory effects, including regulation of T-cell differentiation and cytokine production [5, 6]. These properties make RAD001 of particular interest in studies exploring its potential role in modulating antitumor immune responses.

Influenza infection poses a significant threat to immunocompromised individuals, including patients with cancer. Annual influenza vaccination is recommended for patients with cancer. Importantly, accumulating clinical evidence indicates that influenza vaccination can be safely administered to patients receiving ICIs. Retrospective studies have reported no significant increase in the incidence of immune-related adverse events (irAEs) among influenza-vaccinated patients treated with PD-1/PD-L1 inhibitors compared with unvaccinated patients [7–9]. However, other studies have raised concerns about a potential increase in irAEs, underscoring the need for more definitive safety data. The specific role of the RAD001 adjuvant in this context, particularly when administered systemically in combination with ICIs, therefore remains insufficiently explored [10].

Vaccines adjuvanted with potent innate immune activators such as RAD001 represent attractive candidates for combination with ICIs. RAD001 has been reported to remodel immunosuppressive cellular components within the TME by reducing Tregs, downregulating myeloid-derived suppressor cells (MDSCs), and modulating tumor-associated macrophages (TAMs) [6, 11]. As a mechanistic comparator, we also included a parallel control using a vaccine adjuvanted with MF59. MF59 is a squalene-based oil-in-water emulsion adjuvant that has been widely used in licensed influenza vaccines. Unlike classical depot-forming adjuvants, MF59 enhances immune responses primarily by inducing a local immunostimulatory environment at the injection site. It promotes the recruitment of antigen-presenting cells (APCs), facilitates antigen uptake, and stimulates the production of chemokines and cytokines, thereby enhancing antigen presentation and subsequent adaptive immune responses. These mechanisms contribute to improved antibody responses and the activation of cellular immunity, making MF59 an effective adjuvant for enhancing vaccine-induced immune response [12, 13].

Despite significant advances in immune checkpoint blockade therapy, a substantial proportion of tumors, particularly those with low PD-L1 expression, remain poorly responsive to anti-PD-1 treatment. Strategies that can enhance antitumor immune responses in this context are therefore of considerable interest. Vaccination-based approaches have the potential to improve antigen-specific immunity and synergize with checkpoint inhibitors; however, the optimal vaccination strategy under PD-1 blockade conditions remains unclear.

This study was therefore designed to address two key questions. First, is the combination of a systemically administered RAD001-adjuvanted influenza vaccine and anti-PD-1 therapy safe in a murine model of lung cancer? Second, beyond safety, can this vaccine enhance the antitumor efficacy of PD-1 blockade by modulating the tumor immune microenvironment?

## Materials and Methods

### Mice and tumor cell line

Female C57BL/6 mice aged 6–8 weeks were obtained from Vital River Laboratories (Beijing, China). Animals were maintained in a specific pathogen-free facility under a 12 h light–dark cycle with *ad libitum* access to food and water. All experimental procedures were conducted in compliance with all the applicable institutional ethical guidelines for the care, welfare and use of animals and approved by the Institutional Animal Care and Use Committee (IACUC) of Ninbo University. The Lewis lung carcinoma (LLC) cell line was obtained from the American Type Culture Collection (ATCC, Manassas, VA, USA). Cells were cultured in Dulbecco’s modified Eagle medium (DMEM; Gibco, Thermo Fisher Scientific, Waltham, MA, USA) supplemented with 10% fetal bovine serum (FBS; Gibco) and 1% penicillin–streptomycin (Gibco). Cells were maintained at 37 °C in a humidified incubator containing 5% CO_2_, as previously described [14].

### Cell line characterization and PD-L1 analysis

The LLC cell line was used to evaluate basal PD-L1 expression by flow cytometry. Cells were detached using 1 mL of 0.25% EDTA-free trypsin (Gibco) and gently pipetted to obtain a single-cell suspension. The cells were then collected by centrifugation at 1,000 rpm for 5 min and washed twice with phosphate-buffered saline (PBS; Gibco) at 500 × g for 5 min. After washing, cells were resuspended in 100 µL of flow cytometry buffer and incubated with Anti-Mouse PD-L1/CD274 antibody (Cat. No. 98166-1-RR; Proteintech, Wuhan, China) at a final amount of 0.25 µg per sample. A live/dead discrimination dye was used before antibody staining to exclude nonviable cells. PD-L1 expression was quantified among viable singlet cells by flow cytometry using fluorescence controls. The incubation was performed at 4 °C for 30 min in the dark. Following staining, cells were washed twice with flow cytometry buffer and resuspended in 400 µL of the same buffer. Samples were immediately analyzed using a flow cytometer equipped with a fluorescein isothiocyanate (FITC) detection channel.

### Tumor inoculation and experimental design

The MF59-adjuvanted influenza vaccine (MF59-Vax) was formulated to contain 3.75 µg of hemagglutinin (HA) derived from the A/California/7/2009 (H1N1)pdm09-like virus. RAD001 (Selleck Chemicals, Houston, TX, USA) was incorporated into the vaccine formulation (RAD001-Vax). RAD001-Vax was administered intraperitoneally together with the vaccine preparation at a dose of 1 mg/kg. Intraperitoneal administration was selected based on previous murine studies demonstrating effective systemic exposure and biological activity of RAD001 [15–18].

To establish the tumor model, 1 × 10^6^ LLC cells suspended in 100 µL of sterile PBS were injected subcutaneously into the right flank of each mouse as previously described [19, 20]. Tumor growth was measured on days 1, 5, 10, and 14 using a digital caliper, and tumor volume was calculated using the formula (length × width^2^)/2. When the mean tumor volume reached approximately 50 mm^3^, mice were randomly assigned to five experimental groups (n = 8 per group) and monitored throughout the 14-day treatment period. Group 1 (control): Intraperitoneal injection of rat IgG2a isotype control antibody (200 µg per dose; Clone 2A3; Bio X Cell, Lebanon, NH, USA) every 3 days. Group 2 (anti-PD-1): Intraperitoneal injection of anti-PD-1 antibody (200 µg per dose; Clone RMP1-14; Bio X Cell) every 3 days. Group 3 (vaccine): Intraperitoneal injection of anti-PD-1 antibody (200 µg per dose; Bio X Cell) administered 3 days before and after vaccination with split influenza vaccine containing 6 µg HA from the A/California/7/2009 (H1N1)pdm09-like virus. Vaccination was performed twice at a 7-day interval. Group 4 (MF59-Vax): Intraperitoneal injection of anti-PD-1 antibody (200 µg per dose; Bio X Cell) administered every 3 days before and after vaccination with MF59-adjuvanted influenza vaccine containing 6 µg HA from the A/California/7/2009 (H1N1)pdm09-like virus and 100 µ MF59 emulsion. Vaccination was performed twice at a 7-day interval. Group 5 (RAD001-Vax): Intraperitoneal injection of anti-PD-1 antibody (200 µg per dose; Bio X Cell) administered every 3 days before vaccination with RAD001-adjuvanted influenza vaccine, with two vaccinations given at a 7-day interval. A treatment timeline schematic has been added here (Supplementary Material 1, wjon.elmerpub.com).

### Safety and tolerability assessment

Mice were monitored daily for signs of treatment-related toxicity, including ruffled fur, lethargy, diarrhea, and mortality. At the experimental endpoint (day 14 or when tumor volume exceeded 1,500 mm^3^), mice were euthanized, and major organs (liver, lungs, heart, kidneys, and intestines) of a subset of mice (n = 4 per group) were harvested for histopathological evaluation. The tissues were fixed in 10% neutral-buffered formalin, embedded in paraffin, and sectioned at a thickness of 5 µm. Tissue sections were stained with hematoxylin and eosin (H&E) and examined by a pathologist blinded to the treatment groups to assess evidence of immune cell infiltration or tissue damage.

### Sample collection and immune cell analysis

On day 14 after initiation of anti-PD-1 antibody treatment, mice (n = 8 per group) were euthanized for immune profiling. Blood samples were collected by cardiac puncture and processed for serum separation. Paraffin-embedded tumor tissue sections were deparaffinized and subjected to heat-mediated antigen retrieval. Endogenous peroxidase activity was quenched using 0.3% hydrogen peroxide (H_2_O_2_) in methanol. Sections were then blocked with 3% bovine serum albumin (BSA) in PBS for 1 h at room temperature.

The sections were incubated overnight at 4 °C with primary antibodies against Foxp3 (1:400; Cat. No. 12653S; Cell Signaling Technology, Danvers, MA, USA), CD4 (1:400; Cat. No. ab183685; Abcam, Cambridge, MA, USA), or CD8 (1:400; Cat. No. ab217344; Abcam). After incubation with horseradish peroxidase (HRP)-conjugated secondary antibodies, signal amplification was performed using a tyramide signal amplification (TSA) kit (Haokebio, Hangzhou, China). The antibody staining cycle was repeated three times to enable multiplex detection. Nuclei were subsequently counterstained with 4′,6-diamidino-2-phenylindole dihydrochloride (DAPI) [21, 22]. Stained sections were visualized using a Nikon ECLIPSE C1 fluorescence microscope (Nikon Instruments Inc., Tokyo, Japan) at × 200 and × 400 magnifications [21]. Spectral unmixing was performed using inForm software (version 2.4; Akoya Biosciences, Marlborough, MA, USA) to remove tissue autofluorescence and correct for fluorophore spectral overlap. Quantitative analysis of cellular co-expression patterns was conducted using the HALO™ image analysis platform (Indica Labs, Albuquerque, NM, USA).

### Hemagglutination inhibition (HI) assay

Prior to analysis, all serum samples were treated with receptor-destroying enzyme (RDE; Denka Seiken Co., Tokyo, Japan) to eliminate nonspecific inhibitors [22]. For the HI assay, two-fold serial dilutions of serum samples were incubated with four hemagglutinating units of A(H1N1)pdm09 virus at 37 °C for 1 h. Subsequently, 50 µL of 1% chicken erythrocytes (Solarbio Life Sciences, Beijing, China) were added to each well, and the mixtures were incubated at 4 °C for 1 h. Hemagglutination patterns were then visually assessed, and HI titers were determined within 10 min.

### Microneutralization (MN) assay

Madin–Darby canine kidney (MDCK) cells (ATCC) were seeded into 96-well plates at a density of 2 × 10^4^ cells per well and cultured until reaching approximately 80–90% confluence at 37 °C in a humidified incubator. Heat-inactivated serum samples were initially diluted 1:10 in DMEM (Gibco), followed by two-fold serial dilutions. Each diluted serum sample was then mixed with 50 µL containing 100 median tissue culture infectious doses (TCID_50_) of A(H1N1)pdm09 virus and incubated at 37 °C for 1 h to allow virus–antibody interaction. The serum–virus mixtures were subsequently added to MDCK cell monolayers and incubated for 72 h at 37 °C in culture medium supplemented with TPCK-treated trypsin (Sigma-Aldrich, St. Louis, MO, USA). After incubation, culture supernatants were collected and transferred to V-bottom 96-well plates. Viral replication was assessed by hemagglutination using chicken erythrocytes.

### Cytokine analysis

Serum cytokine concentrations, including interferon-γ (IFN-γ), interleukin (IL)-2, IL-12p70, IL-4, IL-10, granulocyte colony-stimulating factor (G-CSF), IL-17, growth-related oncogene alpha (GROα), IL-1β, IL-23p19, IL-6, monocyte chemoattractant protein-1 (MCP-1), and tumor necrosis factor-α (TNF-α), were quantified using RayBio^®^ RayPlex cytokine assay kits (Cat. No. FAM-INF-1-48; RayBiotech, Peachtree Corners, GA, USA) according to the manufacturer’s instructions. Briefly, diluted serum samples or controls (25 µL) were mixed with 25 µL of assay buffer and 25 µL of bead–antibody complexes, followed by incubation at room temperature for 2 h with gentle shaking. After washing, plates were incubated with biotinylated detection antibodies (25 µL per well) for 1 h at room temperature. Subsequently, 50 µL of streptavidin–phycoerythrin (streptavidin-PE) was added and incubated for an additional 30 min. The plates were then washed, and the beads were resuspended in 150 µL of wash buffer for analysis [23]. The 96-well plates were analyzed using a compatible fluorescence detection system, and cytokine concentrations were calculated based on standard curves generated for each analyte. For samples in which cytokine levels were below the detection limit, the value corresponding to the lowest standard concentration was used for statistical analysis.

### Transcriptome sequencing

Strand-specific RNA libraries were constructed from mRNA isolated from total RNA using poly-T magnetic beads. Second-strand complementary DNA (cDNA) synthesis was performed with incorporation of dUTP to maintain strand specificity. After library quality assessment, indexed libraries were pooled according to their effective concentrations and the desired sequencing depth, and sequencing was performed on an Illumina platform (Illumina Inc., San Diego, CA, USA). For downstream bioinformatic analysis, clean sequencing reads were aligned to the reference genome using HISAT2 (version 2.2.1). Transcript assembly was performed using StringTie (version 2.2.3) based on the reference genome annotation [24], and gene-level read counts were obtained using FeatureCounts (version 2.0.6). Gene expression levels were quantified as fragments per kilobase of transcript per million mapped reads (FPKM). Differential gene expression analysis between experimental groups was performed using DESeq2 with biological replicates. Genes with an adjusted P value (Padj) ≤ 0.05 and an absolute log2 fold change ≥ 1 were considered significantly differentially expressed. Gene Ontology (GO) and Kyoto Encyclopedia of Genes and Genomes (KEGG) pathway enrichment analyses were subsequently performed for the identified differentially expressed genes.

### Statistical analysis

All statistical analyses were performed using GraphPad Prism (version 9.0; GraphPad Software, San Diego, CA, USA). Tumor growth curves over time were analyzed using two-way repeated-measures analysis of variance (ANOVA). Comparisons of flow cytometry data, cytokine levels, and other continuous variables among multiple groups were performed using one-way ANOVA followed by Tukey’s *post hoc* test for multiple comparisons. Data are presented as the mean ± standard error of the mean (SEM). A P value < 0.05 was considered statistically significant.

## Results

### PD-L1 expression and combination therapy safety

Flow cytometry analysis confirmed that LLC cells exhibited low basal PD-L1 expression, with 10.28% of cells identified as PD-L1-positive (Supplementary Material 2, wjon.elmerpub.com). Based on commonly used clinical classification, PD-L1 expression of 1–49% is considered low expression, whereas ≥ 50% is defined as high expression [25].

**Figure 1:**
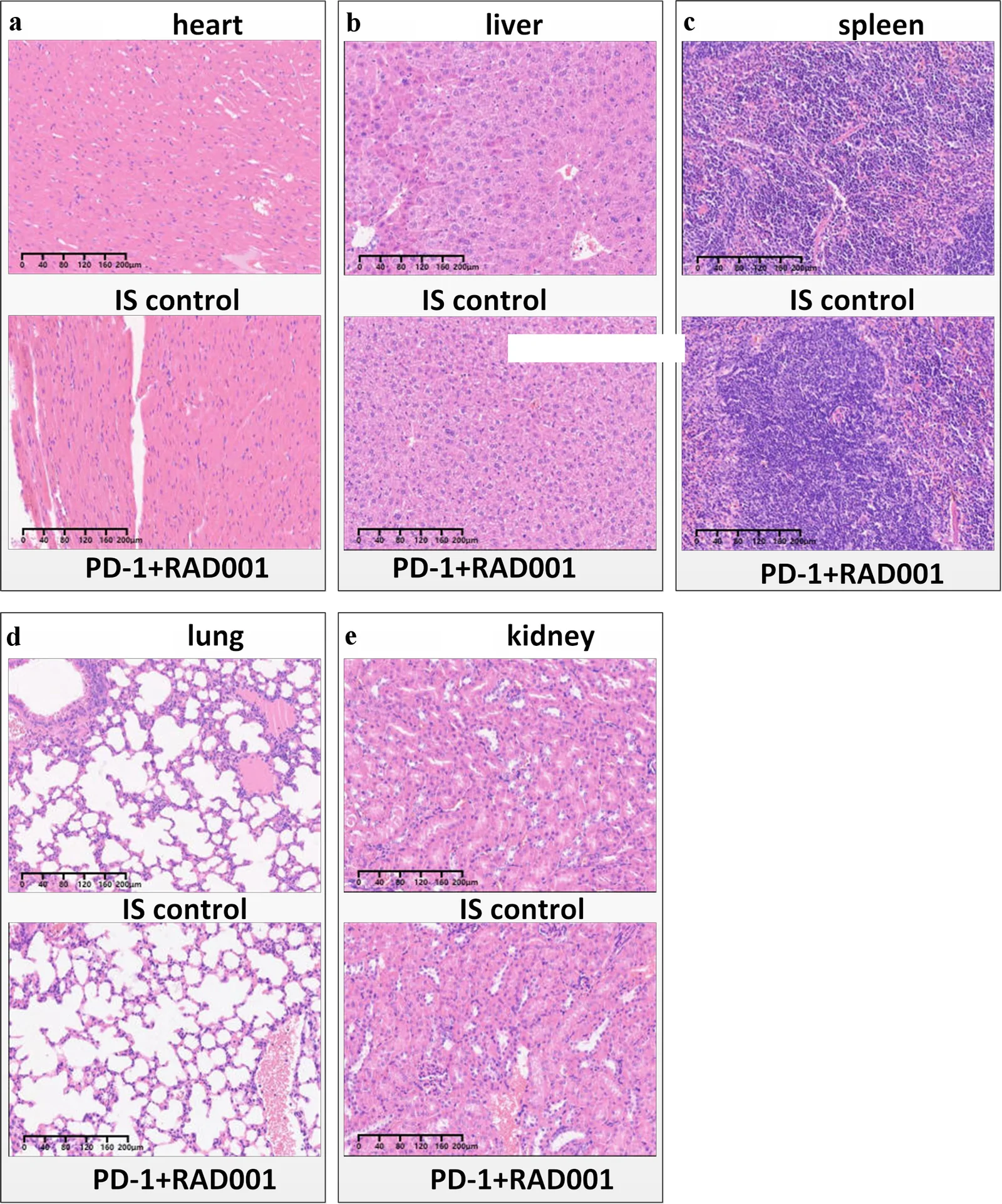
Safety profile of combination therapy. Representative H&E stained sections of heart (a) liver (b), spleen (c) lung (d), and kidney (e) tissues from mice in the RAD001-Vax combination treatment group and control groups at the end of the study are shown. IS control stands for the isotype control. No significant histopathological abnormalities or evidence of immune-mediated tissue damage were observed. H&E: hematoxylin and eosin.

A major safety concern when combining an immune-stimulating vaccine with ICIs is the potential exacerbation of irAEs. In the present study, no animals in any treatment group reached a moribund state or required premature euthanasia due to treatment-related toxicity. Due to ethical considerations, mice were euthanized when tumor volume reached 1,500 mm^3^ in accordance with institutional animal care guidelines. As a result, no spontaneous mortality events occurred during the study period, and conventional survival analysis was not applicable in this model. Histopathological examination of major organs, including the liver, lungs, heart, kidneys, and spleen, revealed no significant evidence of immune-mediated tissue damage, such as leukocyte infiltration or tissue necrosis, in the combination therapy groups compared with the monotherapy or control groups (Fig. 1). To further evaluate systemic safety, additional biochemical analyses were performed using stored serum samples. No obvious abnormalities were observed in routine serum biochemical analysis, including alanine aminotransferase (ALT), aspartate aminotransferase (AST), creatinine, and other biochemical parameters showed no significant abnormalities among treatment groups (Supplementary Material 3, wjon.elmerpub.com). These findings indicate that the addition of RAD001-adjuvanted or MF59-adjuvanted influenza vaccine did not exacerbate the organ-specific toxicity profile of anti-PD-1 therapy in this model.

### Evaluation of antibody responses

**Figure 2:**
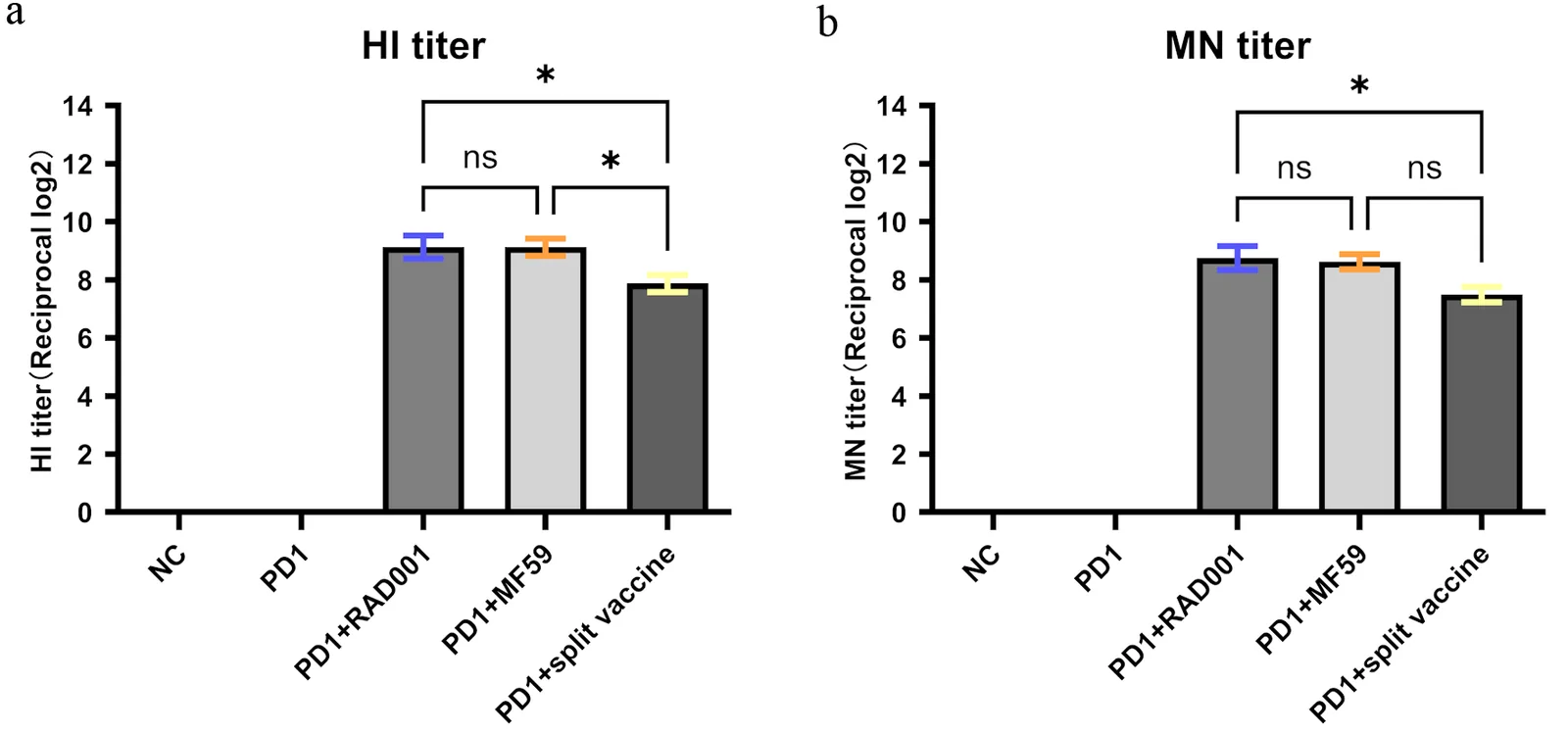
Antibody responses induced by different vaccine formulations. Hemagglutination inhibition (HI) (a) and microneutralization (MN) (b) antibody titers were determined for all five experimental groups. Data are presented as geometric mean titers (GMT) for each group (n = 8 mice per group). Mice received intramuscular injections of the following formulations: group 1, rat IgG2a isotype control antibody; group 2, anti-PD-1 antibody; group 3, anti-PD-1 antibody plus split influenza vaccine; group 4, anti-PD-1 antibody plus MF59-adjuvanted split influenza vaccine; and group 5, anti-PD-1 antibody plus RAD001-adjuvanted split influenza vaccine. *Indicates a statistically significant difference (P < 0.05), and ns indicates no significant difference (P ≥ 0.05). PD-1: programmed cell death protein 1; NC: negative control; IgG: immunoglobulin G.

The lower limit of detection was defined as 1:10 for HI assay and MN assay. Samples below the detection threshold were assigned half of the lowest detectable dilution for calculation of geometric mean titers. Following the second immunization, the RAD001 adjuvant significantly increased both HI and MN antibody titers compared with the split influenza vaccine alone (HI titer: P = 0.0256; MN titer: P = 0.035) (Fig. 2). In contrast, no significant differences were observed between mice receiving the RAD001 adjuvant and those receiving the MF59 adjuvant (HI titer: P > 0.9; MN titer: P = 0.803).

### RAD001-adjuvanted vaccine synergizes with anti-PD-1 to suppress tumor growth and improve survival

**Figure 3:**
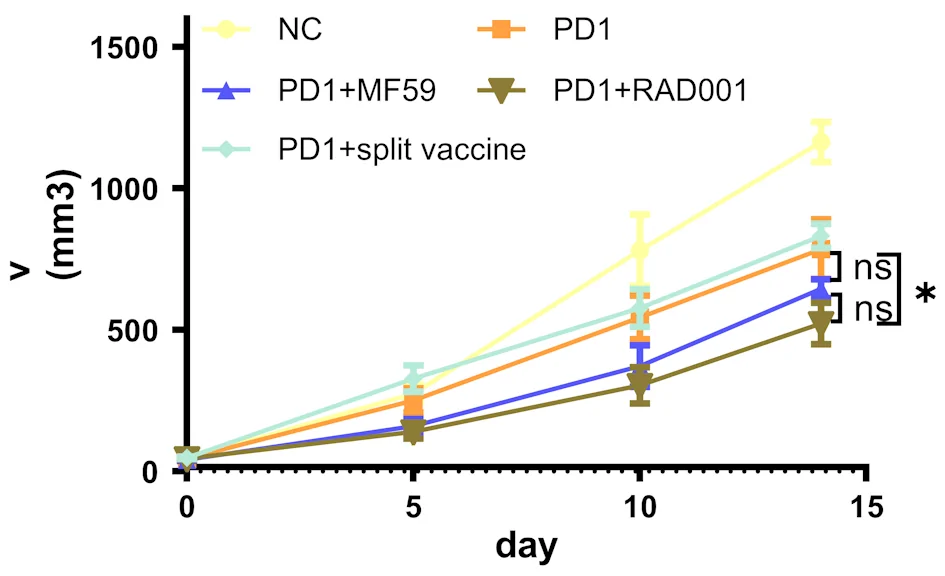
Tumor growth kinetics in mice treated with different vaccine formulations. Tumor volumes were measured in five experimental groups: control, anti-PD-1, anti-PD-1 + split vaccine, anti-PD-1 + RAD001, and anti-PD-1 + MF59. Day 0 represents the first vaccine administration, and tumor growth is presented relative to this time point. Data are shown as mean ± SEM with individual measurements indicated. Statistical analysis was performed using two-way repeated-measures ANOVA followed by Tukey’s multiple comparisons test. Differences between groups are presented as indicated. *Indicates a statistically significant difference (P < 0.05), and ns indicates no significant difference (P ≥ 0.05). NC: negative control; PD-1: programmed cell death protein 1; SEM: standard error of the mean; ANOVA: analysis of variance.

In the LLC tumor model, tumor growth curves were generated to evaluate the therapeutic efficacy of PD-1 blockade, split influenza vaccine, and the combinations of PD-1 blockade with MF59- or RAD001-adjuvanted split influenza vaccine. As shown in Figure 3, all groups exhibited progressive tumor enlargement during the observation period; however, the rate of tumor growth varied among the different treatment groups. Mice treated with PD-1 blockade alone showed a significant delay in tumor progression compared with the control group, indicating that PD-1 inhibition effectively suppressed tumor growth. The combination of PD-1 blockade and RAD001-adjuvanted vaccine produced a more pronounced and sustained suppression of tumor growth. Mean tumor volumes in this group remained approximately 33% smaller than those in the PD-1 monotherapy group from day 14 onward, tumor growth was analyzed using two-way repeated-measures ANOVA with Greenhouse-Geisser correction (ε = 0.6865), followed by Tukey’s multiple-comparisons test. At day 14, tumor volumes were significantly lower in the anti-PD-1 + RAD001 group than in the anti-PD-1 monotherapy group (Tukey-adjusted P = 0.014). The corresponding ANOVA statistics were F(1,14) = 7.881, with df = 1. suggesting that RAD001 enhanced the therapeutic efficacy of PD-1 blockade. These findings demonstrate a synergistic antitumor effect between PD-1 blockade and the RAD001-adjuvanted vaccine. This combined treatment likely enhances the priming and activation of tumor-specific T cells, thereby promoting a stronger and more sustained immune-mediated suppression of tumor growth.

### Combination therapy remodels the tumor immune microenvironment

**Figure 4:**
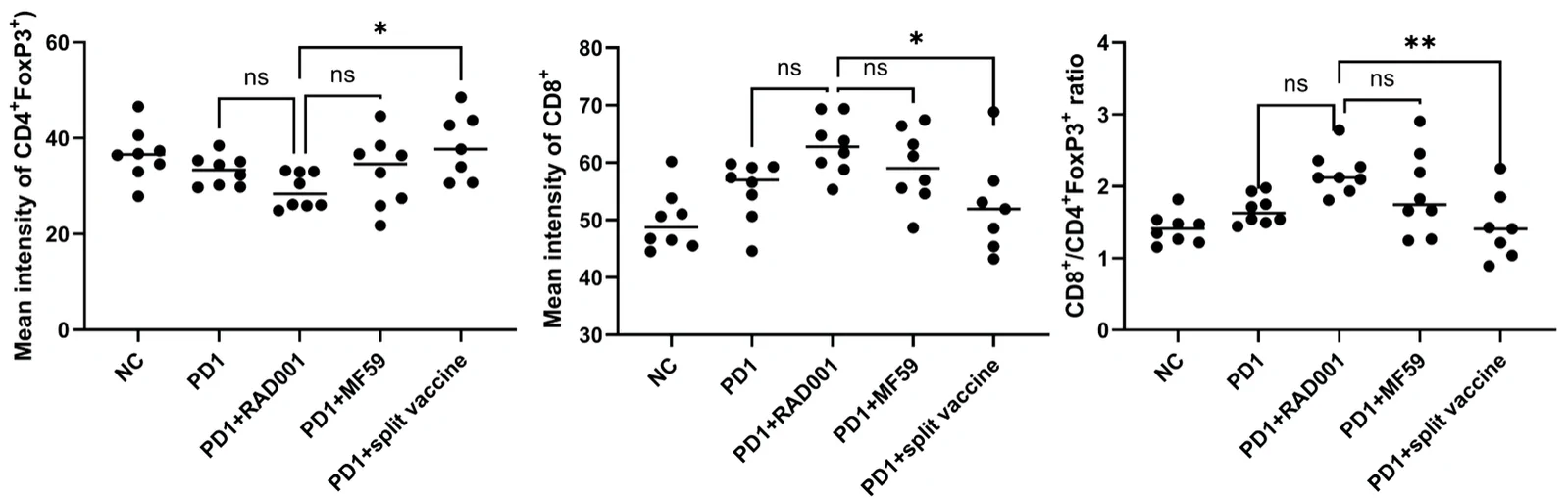
Immunophenotyping of tumor-infiltrating lymphocytes (day 14). The percentage of regulatory T cells (Tregs; FoxP3^+^ within CD4^+^ T cells; left), CD8^+^ T cells (middle), and the CD8^+^/Treg ratio (right) are shown. Data are presented as mean ± SEM (n = 8 mice per group), with individual values indicated. *Indicates a statistically significant difference (P < 0.05), **P < 0.01, and ns indicates no significant difference (P ≥ 0.05). SEM: standard error of the mean; NC: negative control; PD-1: programmed cell death protein 1.

**Figure 5:**
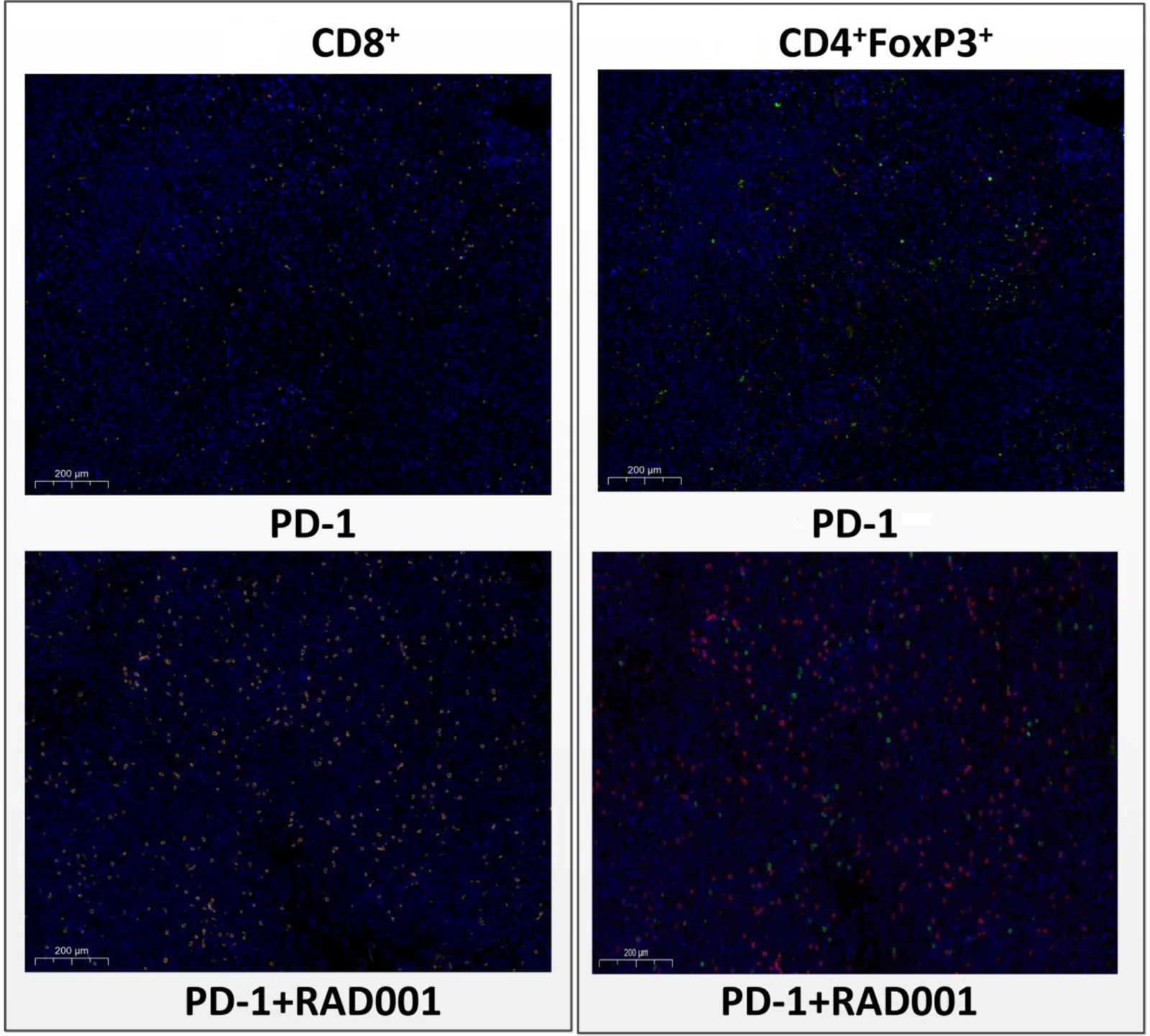
Representative immunofluorescence analysis of tumor-infiltrating lymphocytes (day 14). Representative immunofluorescence images showing DAPI (blue), CD8 (orange), FoxP3 (red), and CD4 (green) staining in tumor tissues from mice treated with anti-PD-1 alone or in combination with RAD001-adjuvanted split influenza vaccine. Differences in immune cell distribution can be observed across treatment groups. PD-1: programmed cell death protein 1.

To investigate the immunological mechanisms underlying the enhanced antitumor efficacy, TILs were analyzed on day 14. Multicolor immunofluorescence analysis revealed that RAD001-based combination therapy increased the infiltration of cytotoxic CD8^+^ T cells while reducing the proportion of immunosuppressive regulatory T cells (CD4^+^FoxP3^+^ Tregs) within the CD4^+^ T-cell population, compared with the PD-1 treatment group (CD8^+^ T cells: P = 0.0095; Tregs: P = 0.0334) (Figs. 4, 5). As a result, the CD8^+^/Treg ratio, an important indicator of effective antitumor immunity, was highest in the RAD001 combination group, exceeding that of the anti-PD-1 monotherapy group by approximately 30% (P = 0.0016) (Fig. 4). These findings indicate that RAD001-based combination therapy effectively remodels the tumor immune microenvironment by enhancing cytotoxic T-cell infiltration while simultaneously reducing immunosuppressive T-cell populations.

### Combination therapy induces a systemic Th1-biased cytokine response

**Figure 6:**
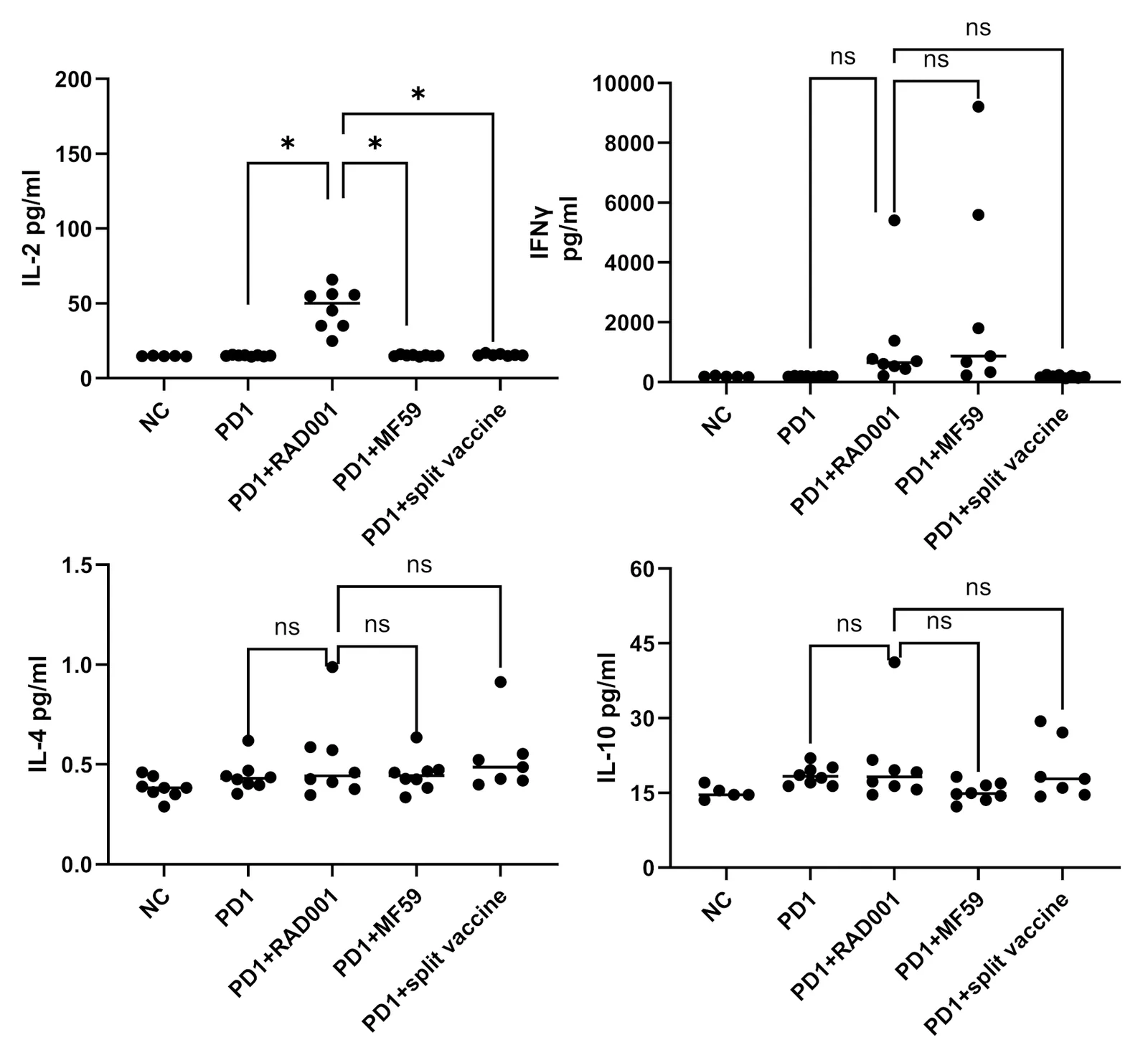
Serum cytokine profile. Serum levels of Th1 cytokines (IFN-γ and IL-2) and Th2-associated immunosuppressive cytokines (IL-4 and IL-10) were measured by ELISA on day 14. Data are presented as mean ± SEM (n = 8 mice per group). *Indicates a statistically significant difference (P < 0.05), and ns indicates no significant difference (P ≥ 0.05). IL: interleukin; IFN: interferon; SEM: standard error of the mean; NC: negative control; ELISA: enzyme-linked immunosorbent assay.

**Figure 7:**
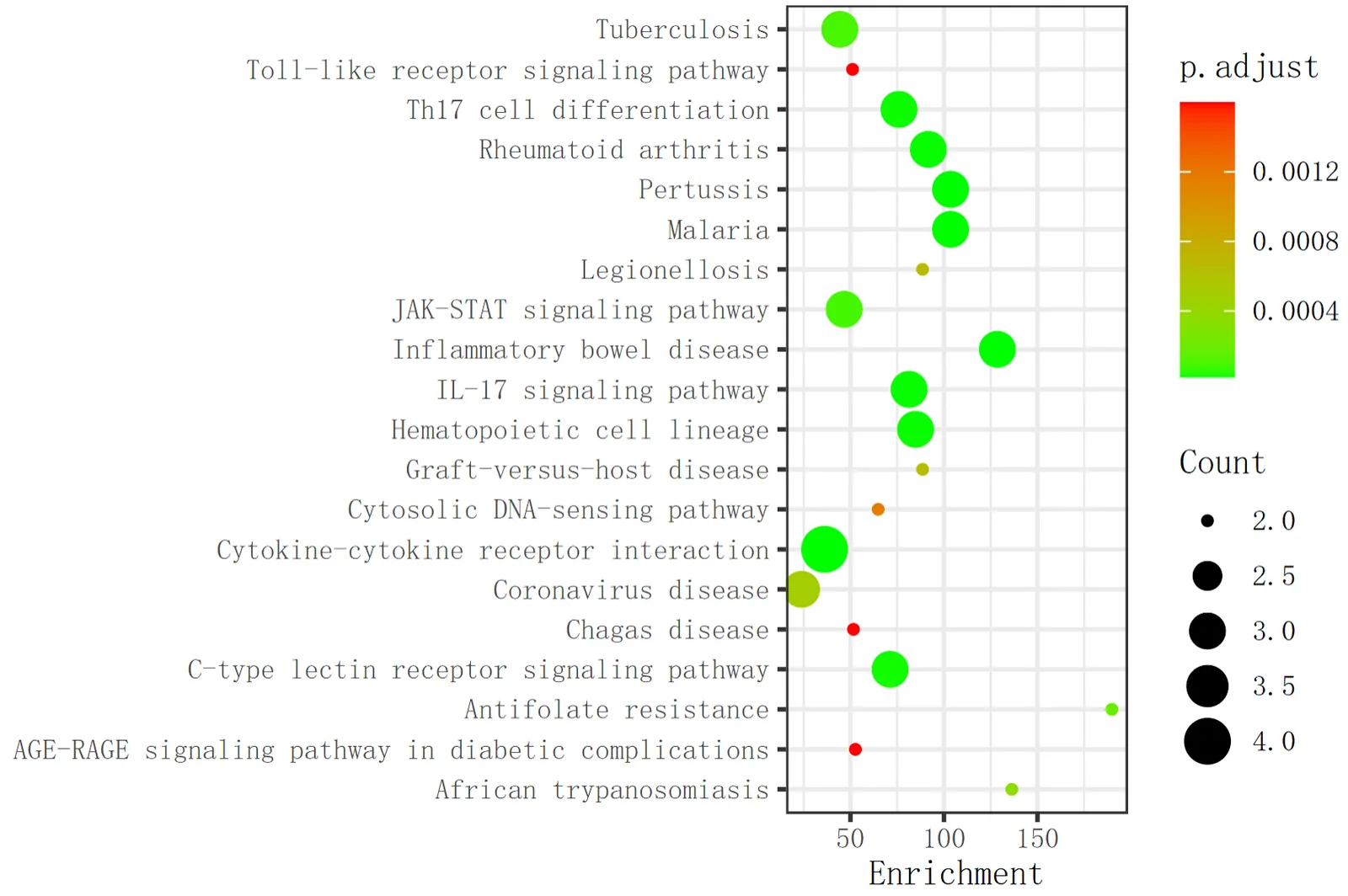
Enriched signaling pathways associated with combination therapy. Pathway enrichment analysis based on cytokine detection results demonstrated that several immune-related signaling pathways were enriched in the PD-1 + RAD001 adjuvanted split vaccine group compared with the PD-1 monotherapy group. PD-1: programmed cell death protein 1.

Serum cytokine analysis further demonstrated that the RAD001-based combination treatment was associated with increased Th1-associated cytokine responses, including a significant increase in IL-2 and a tendency toward increased IFN-γ production (Fig. 6). On day 14, mice receiving the combination therapy exhibited an upward trend in Th1 cytokines IL-2 and IFN-γ compared with the PD-1 treatment group (IFN-γ: P = 0.066; IL-2: P = 0.039), both of which are critical mediators of cytotoxic T-cell activation and cell-mediated immunity. In contrast, the levels of the Th2 cytokine IL-4 and the immunosuppressive cytokine IL-10 remained low and did not differ significantly between groups (IL-4: P = 0.342; IL-10: P = 0.506). These results indicate that the combination therapy promotes a systemic Th1-biased immune response that is associated with effective antitumor immunity. Consistent with this immune activation, pathway enrichment analysis revealed that several immune-related signaling pathways, including the JAK–STAT signaling pathway, cytokine–cytokine receptor interaction, and Th17 cell differentiation, were enriched in the RAD001 combination group compared with the PD-1 monotherapy group (Fig. 7).

### Transcriptomic analysis: KEGG and GO pathway enrichment

**Figure 8:**
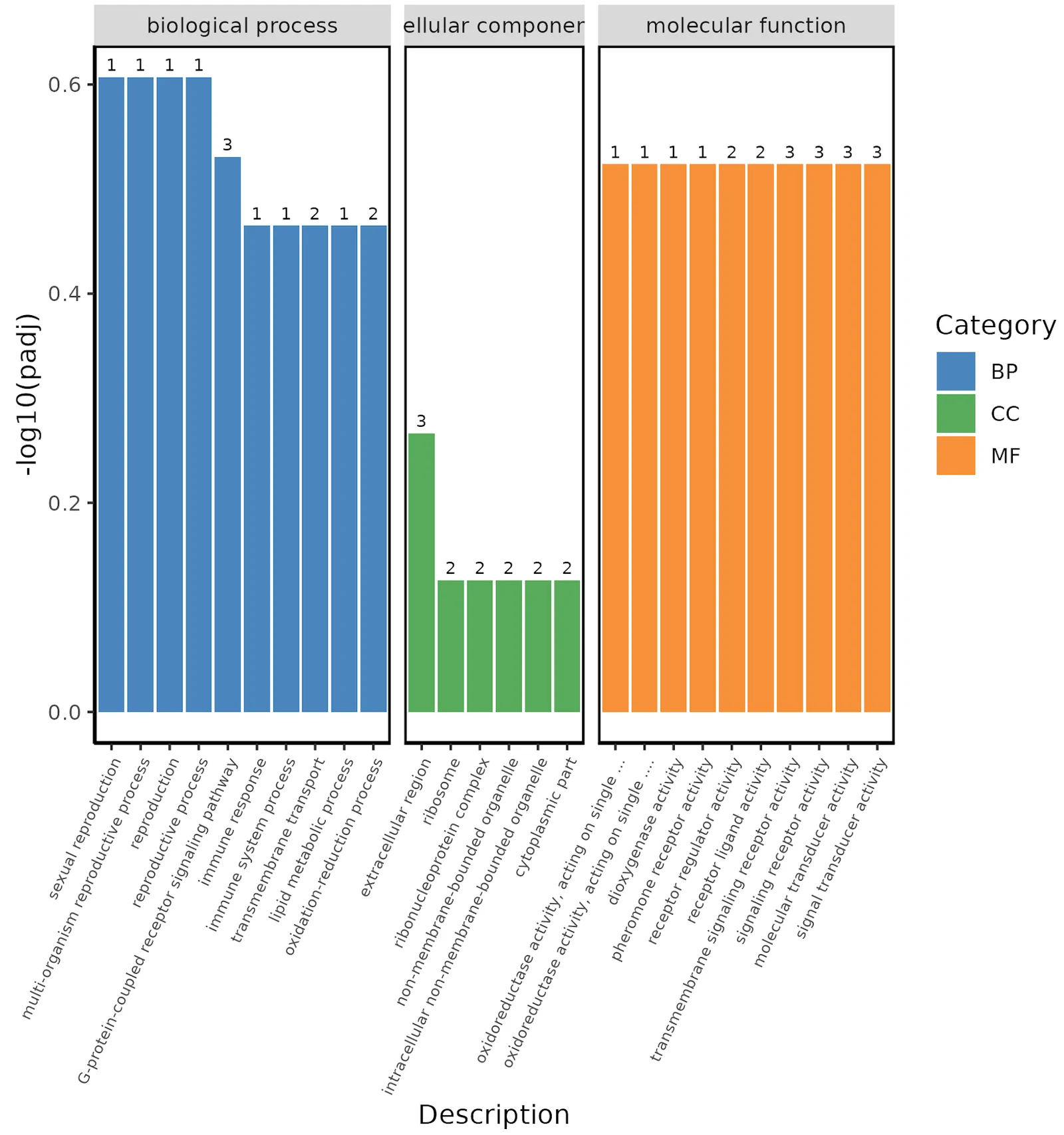
Gene Ontology (GO) enrichment analysis. GO enrichment analysis was performed to categorize differentially expressed genes (DEGs) between the RAD001-adjuvanted vaccine group and the anti-PD-1 therapy group into biological processes (BP), cellular components (CC), and molecular functions (MF).

To further elucidate the molecular mechanisms underlying the synergistic antitumor effect of the RAD001-adjuvanted influenza vaccine combined with anti-PD-1 therapy, transcriptomic analysis was performed. Differential gene expression analysis revealed significant modulation of multiple immune- and tumor-related pathways following combination treatment. GO enrichment analysis identified several significantly enriched biological processes (Fig. 8). Specifically, genes involved in immune processes and immune system processes were upregulated, including pathways associated with T-cell activation, cytokine production, and antigen presentation. These findings suggest that the combined therapy may enhance immune surveillance and antitumor immune responses. In addition, pathways related to G protein–coupled receptor (GPCR) signaling were significantly enriched. GPCR-associated receptor regulatory and ligand activities are known to contribute to tumor progression and metastasis through modulation of immune responses. Because GPCRs can be activated by a wide range of ligands, including neurotransmitters, chemokines, and sensory stimuli, these pathways may represent potential targets for cancer immunotherapy [26, 27].

**Figure 9:**
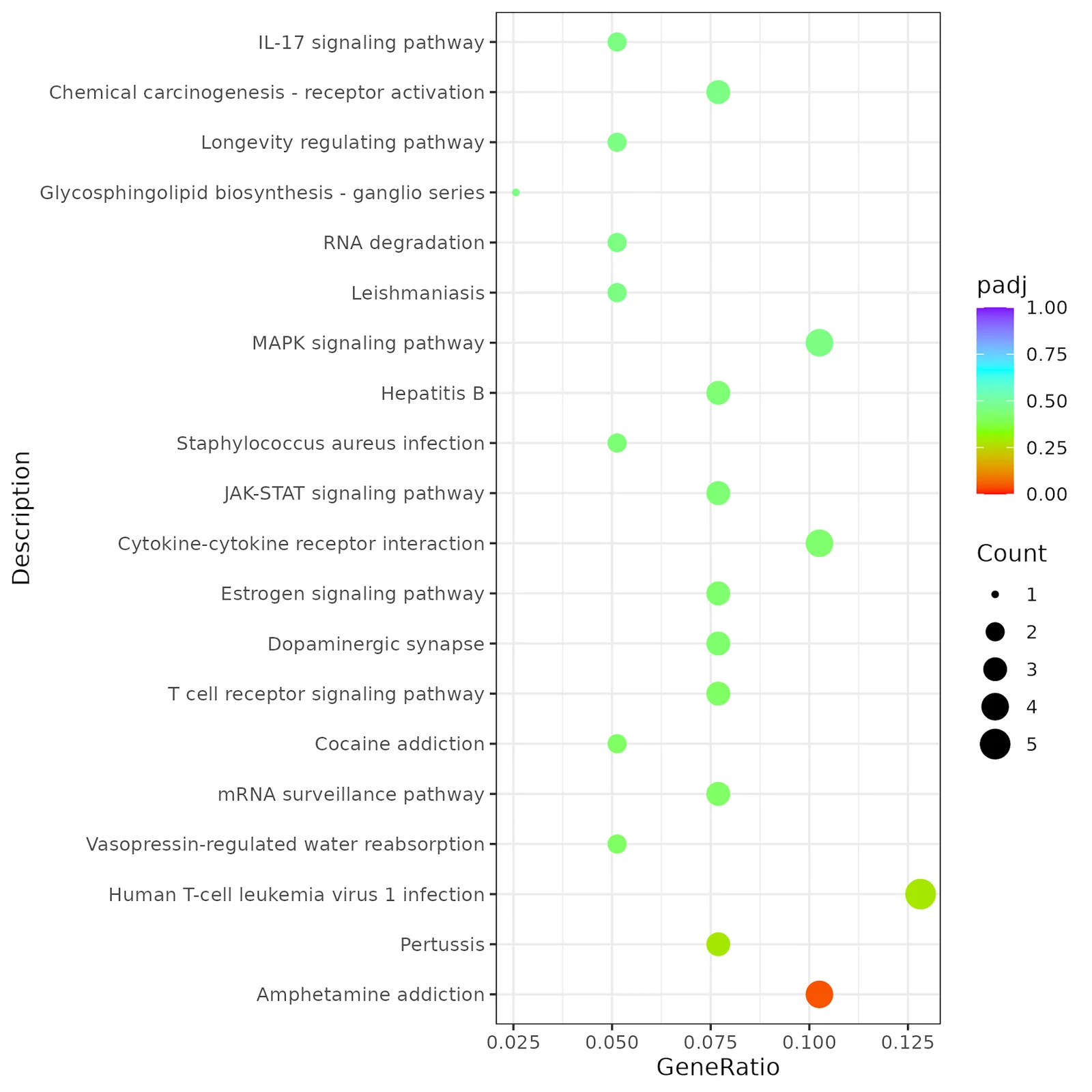
Kyoto Encyclopedia of Genes and Genomes (KEGG) pathway analysis. KEGG enrichment analysis was conducted to identify the top 20 signaling pathways enriched among differentially expressed mRNAs between the RAD001-adjuvanted influenza vaccine group and the anti-PD-1 therapy group. PD-1: programmed cell death protein 1.

KEGG pathway enrichment analysis further identified several critical signaling pathways modulated by RAD001-based combination therapy (Fig. 9). Among these, immune checkpoint–related pathways were significantly activated, including PD-1/PD-L1 signaling and downstream immune activation pathways involving CD8^+^ T-cell responses and IFN-γ signaling. These results support the hypothesis that the RAD001-adjuvanted influenza vaccine enhances the efficacy of PD-1 blockade by strengthening immune checkpoint–mediated antitumor responses. Additional enriched pathways included cytokine–cytokine receptor interaction, which reflects the central role of inflammatory cytokines in regulating immune responses and tumor rejection [28, 29]. The T-cell receptor signaling pathway was also significantly upregulated, suggesting enhanced T-cell activation and proliferation [30]. Furthermore, enrichment of the mitogen-activated protein kinase (MAPK) signaling pathway was observed, a pathway known to regulate cellular proliferation, survival, and differentiation, which may further promote immune activation within the TME [31, 32].

## Discussion

This study provides compelling preclinical evidence that administration of an RAD001-adjuvanted influenza vaccine is not only safe but also beneficial when combined with anti-PD-1 therapy in a murine lung cancer model. Notably, no additive toxicity was observed, addressing a concern regarding the combination of nonspecific immune stimulants with ICIs. These findings are consistent with recent retrospective clinical analyses reporting that influenza vaccination did not increase the incidence of irAEs in patients receiving pembrolizumab [33].

The pronounced tumor suppression observed in the PD-1 + RAD001 group suggests a synergistic interaction between checkpoint blockade and adjuvant-mediated immune activation. PD-1 inhibition alone partially restored antitumor immunity by reversing T-cell exhaustion, as reflected by reduced tumor growth compared with the control group. However, the combination with RAD001 produced a more pronounced and sustained inhibitory effect, indicating that the adjuvant enhances PD-1–mediated immune responses rather than acting independently. The RAD001-adjuvanted vaccine appears to function as a potent immune modulator, rendering the TME more permissive to PD-1 blockade. The observed increase in activated cytotoxic CD8^+^ T cells and the favorable shift in the CD8^+^/Treg ratio represent key hallmarks of effective antitumor immunity [34, 35]. This mechanism differs from that of MF59, which primarily promotes T-cell priming through recruitment and activation of APCs. By enhancing cytotoxic T-cell infiltration and reducing immunosuppressive elements within the TME, the RAD001-based strategy may enrich cytotoxic T cells [36, 37], thereby overcoming a mechanism of resistance to ICIs.

Our results demonstrate that even systemic intraperitoneal administration can exert meaningful antitumor effects when the vaccine contains a potent adjuvant such as RAD001. This finding suggests that the adjuvant component may play a critical role in inducing the nonspecific immune activation required for effective antitumor responses. The observed shift toward a systemic Th1 cytokine profile, characterized by a trend toward increased IL-2 and IFN-γ levels, may be relevant, as Th1-dominant immune responses are associated with effective antitumor immunity and are considered positive predictors of responsiveness to ICIs, mTOR signaling has context-dependent effects on T-cell immunity, mTORC1 inhibition can promote memory CD8^+^ T-cell differentiation and metabolic reprogramming [5], potentially enhancing the durability and recall capacity of vaccine-induced antitumor responses. However, these cytokine changes cannot be attributed solely to mTOR inhibition, and direct mechanistic studies are required to confirm this interpretation.

Our study provides two key findings. First, we demonstrate that the RAD001-adjuvanted influenza vaccine represents a safe and effective partner for anti-PD-1 therapy, capable of inducing synergistic antitumor immunity in a PD-L1–low lung cancer model. These results raise the intriguing possibility that routine influenza vaccination may confer additional oncological benefits for patients undergoing ICI therapy. Second, by incorporating the MF59-adjuvanted vaccine as a mechanistic comparator, we provide insights into why RAD001 produces superior immunotherapeutic effects. The contrast between the outcomes, synergy with RAD001 versus only additive effects with MF59, highlights that not all adjuvants function equivalently in the context of cancer immunotherapy. RAD001, through its mTOR-inhibitory activity, may reshape immune regulation and enhance immune activation in a manner that amplifies the effects of PD-1 blockade. In contrast, MF59, a squalene-based oil-in-water emulsion, primarily functions by recruiting and activating APCs, particularly dendritic cells (DCs), at the site of antigen exposure. Through potent activation of innate immunity, MF59 can enhance antigen presentation and T-cell priming, thereby contributing to improved immune responses and creating a microenvironment that is more permissive to PD-1 blockade. RAD001 has been extensively investigated in preclinical mouse models using different administration routes. Although oral RAD001 is commonly used clinically, intraperitoneal administration provides a reproducible systemic exposure in experimental animals [15–18].

The immunological effects of mTOR inhibition are complex and highly dependent on dose, timing, and cellular context. Although sustained or high-level mTOR inhibition can suppress T-cell proliferation and effector function, partial mTORC1 inhibition has been shown to promote memory CD8^+^ T-cell differentiation and improve the quality and durability of antigen-specific immune responses. In particular, pharmacological inhibition of mTOR with rapamycin can enhance memory CD8^+^ T-cell formation and recall responses, indicating that mTOR inhibition does not necessarily result in generalized immunosuppression [5]. mTOR signaling also regulates T-cell metabolic programming and differentiation, providing a mechanistic basis for the context-dependent effects of mTOR inhibitors on antitumor immunity [38–40]. In the present study, low-dose RAD001 was administered in combination with influenza vaccination and PD-1 blockade rather than as an immunosuppressive monotherapy. Under this setting, RAD001-mediated mTOR modulation may favor T-cell differentiation and immune remodeling, while vaccination provides immune stimulation and PD-1 blockade releases inhibitory signaling.

Several limitations of the present study should be acknowledged. First, although RAD001 is a well-characterized mTOR inhibitor, direct evidence of mTOR pathway inhibition, such as phosphorylated mTOR (Ser2448) and downstream signaling in tumor tissues, was not assessed. Because mTOR is broadly expressed in both tumor and immune cells, RAD001 may exert both tumor-intrinsic and immunomodulatory effects. Future studies using another mTOR inhibitor, such as temsirolimus (CCI-779), together with cell-type-specific approaches, are warranted to clarify the contribution of mTOR signaling and distinguish these compartment-specific effects. Second, the study was limited to a single subcutaneous LLC model, which does not fully recapitulate the anatomical and immune microenvironment of orthotopic lung tumors; validation in additional and more clinically relevant models is therefore required. Third, immune-cell characterization was primarily based on immunofluorescence, and complementary flow cytometric and functional analyses would strengthen immune profiling. Fourth, although serum biochemical parameters were evaluated, complete blood counts were not assessed and should be incorporated into future safety studies. Finally, mTOR is widely expressed in tumor and immune cells, RAD001 may exert complex effects involving both tumor-intrinsic signaling and immune-cell regulation. Future studies should distinguish these compartment-specific effects using cell-type-specific approaches.

### Conclusions

In conclusion, our findings support the safety and feasibility of administering RAD001-adjuvanted influenza vaccines to lung cancer patients receiving PD-1/PD-L1 inhibitor therapy. Importantly, these results suggest that routine influenza vaccination may not only protect against infection but may also beneficially enhance the efficacy of cancer immunotherapy. The present findings therefore provide a rationale for prospective clinical trials to formally evaluate this therapeutic strategy in patients. Such studies should carefully assess the incidence of irAEs, vaccine immunogenicity, and clinically relevant oncologic outcomes, including objective response rates and progression-free survival.

The RAD001-adjuvanted influenza vaccine represents a promising and clinically translatable strategy for enhancing and broadening the therapeutic efficacy of anti-PD-1 therapy. Its synergistic activity differs from that of adjuvants with distinct mechanisms such as MF59. mTOR inhibition exerts context-dependent immunomodulatory effects rather than purely immunosuppressive functions. It can enhance antitumor immunity by promoting CD8^+^ T-cell memory formation and supporting Th1-biased responses under specific conditions. This combination approach provides a rational strategy to overcome tumor immune resistance and may offer important translational potential for the development of next-generation vaccine–checkpoint inhibitor regimens in cancer immunotherapy.

## Supplementary Material

Suppl 1 Treatment timeline schematic.

Suppl 2 Flow cytometric analysis of PD-L1 surface expression.

Suppl 3 Biochemical analysis of serum samples for safety evaluation.

## Data Availability

The datasets generated or analyzed during the current study are available from the corresponding author upon reasonable request.
