# Supplementary material for: RAD001-Adjuvanted Influenza Vaccine Synergizes With Anti–Programmed Cell Death Protein 1 Therapy to Enhance Antitumor Immunity in Programmed Cell Death Ligand 1–Low Murine Lung Cancer
Source: World J Oncol. 2026 Sep 4;17(5):751–64. doi: 10.14740/wjon2831 (PMC13568780; doi:10.14740/wjon2831)
Supplement: Supplementary file 1 [file wjon-17-05-751-s001.docx]

**Suppl 1.** Treatment timeline schematic. LLC tumor-bearing mice were randomized into five groups when tumor volume reached approximately 50 mm^3^. Anti-PD-1 antibody or IgG2a isotype control antibody was administered intraperitoneally every three days. The first anti-PD-1 administration was performed three days before the first vaccination. Influenza vaccine formulations were administered intraperitoneally on Day 0 and Day 7. Tumor growth was monitored for 14 days after treatment initiation.
