# Supplementary material for: RAD001-Adjuvanted Influenza Vaccine Synergizes With Anti–Programmed Cell Death Protein 1 Therapy to Enhance Antitumor Immunity in Programmed Cell Death Ligand 1–Low Murine Lung Cancer
Source: World J Oncol. 2026 Sep 4;17(5):751–64. doi: 10.14740/wjon2831 (PMC13568780; doi:10.14740/wjon2831)
Supplement: Supplementary file 2 [file wjon-17-05-751-s002.docx]

**Suppl 2.** Flow cytometric analysis of PD-L1 surface expression. Surface expression of PD-L1 on LLC cells was evaluated by flow cytometry using a specific antibody (Proteintech, Cat. No. 98166-1-RR). Following standard staining procedures, cells were analyzed on a flow cytometer, and PD-L1 positivity was determined to be 10.28% after normalization to the isotype control.
