# Supplementary material for: RAD001-Adjuvanted Influenza Vaccine Synergizes With Anti–Programmed Cell Death Protein 1 Therapy to Enhance Antitumor Immunity in Programmed Cell Death Ligand 1–Low Murine Lung Cancer
Source: World J Oncol. 2026 Sep 4;17(5):751–64. doi: 10.14740/wjon2831 (PMC13568780; doi:10.14740/wjon2831)
Supplement: Supplementary file 3 [file wjon-17-05-751-s003.docx]

**Suppl 3.** Biochemical analysis of serum samples for safety evaluation. Serum biochemical parameters were measured in tumor-bearing mice from each treatment group to assess potential systemic toxicity. Data are presented as mean ± SEM (n = 8 mice per group). No obvious abnormalities were observed across treatment groups.
